# Treatment of sleep‐related eating disorder with suvorexant: A case report on the potential benefits of replacing benzodiazepines with orexin receptor antagonists

**DOI:** 10.1002/pcn5.123

**Published:** 2023-07-17

**Authors:** Kentaro Matsui, Ayano Kimura, Kentaro Nagao, Takuya Yoshiike, Kenichi Kuriyama

**Affiliations:** ^1^ Department of Clinical Laboratory, National Center Hospital National Center of Neurology and Psychiatry Kodaira Tokyo Japan; ^2^ Department of Sleep–Wake Disorders, National Institute of Mental Health National Center of Neurology and Psychiatry Kodaira Tokyo Japan; ^3^ Department of Psychiatry, National Center Hospital National Center of Neurology and Psychiatry Kodaira Tokyo Japan

**Keywords:** benzodiazepine, night eating syndrome, orexin receptor antagonist, sleep‐related eating disorder, suvorexant

## Abstract

**Background:**

Nocturnal eating behavior in patients with sleep‐related eating disorder (SRED) is difficult to control and can become chronic, causing weight gain and psychological distress. Here, we report a case of SRED comorbid with major depressive disorder successfully treated by switching from brotizolam to suvorexant, that is, from a benzodiazepine to an orexin receptor antagonist.

**Case Presentation:**

A 25‐year‐old woman complained of night snacking with partial/complete amnesia and sleepwalking for 1 year. She had a diagnosis of major depressive disorder at age 20 and was on paroxetine and brotizolam for depression and insomnia. At 24 years of age, she experienced her second depressive episode, then her amnestic nocturnal eating became prominent. Even after improvement in depressive symptoms, she experienced uncontrollable nocturnal eating episodes every 2 days, resulting in weight gain of over 10 kg. After a partial amnestic eating episode following an awakening from stage N2 sleep was confirmed through video polysomnography, she was diagnosed with SRED. Considering her strong desire to resolve involuntary eating, we instructed her to discontinue brotizolam and start suvorexant. Subsequently, her nocturnal eating completely disappeared. She experienced rebound insomnia, which improved within 1 month. She was then continued on 10 mg of suvorexant and has not experienced nocturnal eating for 2 years.

**Conclusion:**

This case highlights the importance of discontinuing benzodiazepines in the treatment of SRED, but also suggests the potential benefit of orexin receptor antagonists in the treatment of SRED. The efficacy of orexin receptor antagonists in idiopathic SRED should be tested in future studies.

## Graphical abstract

This case study describes a 25‐year‐old woman with sleep‐related eating disorder (SRED) and major depressive disorder who was successfully treated by switching from brotizolam to suvorexant, an orexin receptor antagonist. The patient's nocturnal eating ceased, and she remained symptom‐free for 2 years, suggesting the potential benefit of orexin receptor antagonists in treating SRED.

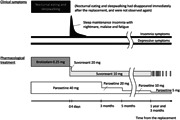

## BACKGROUND

Sleep‐related eating disorder (SRED) is characterized by eating behaviors during sleep accompanied by complete or partial amnesia.^1^, ^2^ It is a type of parasomnia in which food ingestion during sleep can be accompanied by sleepwalking.^3^ Uncontrollable nocturnal eating behavior with amnesia often becomes chronic, causing weight gain and severe distress.^1^, ^2^, ^3^ While SRED can occur idiopathically, it can be activated by benzodiazepine receptor agonists, particularly zolpidem.^1^, ^4^, ^5^, ^6^ Memory impairment due to an agonistic effect on gamma‐aminobutyric acid A (GABA_A_) receptors^7^ and sedative properties elevating the arousal threshold^8^, ^9^ are related to activation of nonrapid eye movement parasomnias, including SRED.

Switching from benzodiazepines to ramelteon, a melatonin receptor agonist, may improve SRED symptoms.^10^ However, whether or not orexin receptor antagonists, which do not bind to GABA_A_ receptors similar to ramelteon, could be safely used in patients with SRED is unclear. Here, we report a case of SRED comorbid with major depressive disorder successfully treated with suvorexant, an orexin receptor antagonist,^11^ after switching from the benzodiazepine brotizolam.

## CASE PRESENTATION

Written informed consent was obtained from the patient for publication of this case. A 25‐year‐old woman complained of nocturnal eating with amnesia and sleepwalking for 1 year. She had no apparent childhood experience with nonrapid eye movement sleep (REM) parasomnias, including confusional arousals, sleep terrors, and sleepwalking. She had been diagnosed with major depressive disorder at 20 years of age and was prescribed paroxetine. Simultaneously, she was also prescribed brotizolam for insomnia symptoms. Her nighttime eating onset coincided with the time of diagnosis of major depressive disorder but had been conscious and episodic. At 24 years of age, she took sick leave due to the second depressive episode. From then, her nocturnal eating with partial/complete amnesia became pronounced. She experienced amnestic nighttime eating every 2 days, leading to a weight gain of over 10 kg. On occasion, her parents witnessed her sleepwalking episodes; she might walk to the living room and then drink tea, or turn on the stove, none of which she could recall later. After 9 months, her mood had improved, and she returned to work. However, she continued to experience nocturnal episodes of eating and sleepwalking, which led to her first visit to our outpatient clinic for sleep disorders. She had no drinking or smoking habits and did not use other substances. She was on paroxetine 40 mg and brotizolam 0.25 mg. Her mood symptoms were generally stable, but she occasionally missed work because of fatigue. She complained of sleepiness and fatigue mainly in the morning. However, she slept well and had a regular schedule of going to bed at 11:00 p.m. and waking up at 6:00–7:00 a.m. Blood samples showed no glucose intolerance, electrolyte abnormalities, or hepatic, renal, or thyroid dysfunction. She did not present any symptoms suggestive of restless legs syndrome. A well‐organized sleep–wake schedule ruled out circadian rhythm sleep–wake disorders and central disorders of hypersomnolence, including Kleine–Levin syndrome.

Based on the course of events, we suspected SRED and sleepwalking and conducted polysomnography (PSG) 1 week after the initial visit. We performed PSG using a digital polygraph (Alice 6; Respironics Inc.) with video monitoring. Sleep stages and sleep‐associated events were scored according to the American Academy of Sleep Medicine guidelines.^12^ Her sleep onset time on the day of the examination was 8:30 p.m. Under video observation, she began sleeping in a sitting position at 2:45 a.m. At 3:25 a.m., she transitioned to a supine position for 15 min, then returned to the sitting position, which she maintained for nearly 2 h. At 5:30 a.m., she showed eating behavior by eating a pastry that we had instructed her to place at her bedside. Electroencephalography showed that she woke up from stage N2 sleep and remained awake during the eating behavior without any epileptiform activities. The eating behavior lasted 4 min, after which she immediately lay down and slept again. In the morning, she could partially recall her eating behavior but could not recall sleeping in a sitting position during the night.

She experienced mild sleep‐related breathing events, with an apnea–hypopnea index of 6.2/h, a periodic limb movement index of 13.3/h, and the arousal index of 13.6/h. Other PSG findings were as follows: sleep latency, 9.5 min; total sleep time, 505.5 min; wake after sleep onset (percentage of sleep period time), 12.2%; and sleep efficiency, 86.2%. The proportions of sleep stages were 15.9% for N1, 57.0% for N2, 19.5% for N3, and 7.6% for REM. REM without atonia occurred in 3.5% (81.5 s) of all REMs, without vocalizations or abnormal behaviors.

Based on these results, we diagnosed her with SRED, sleepwalking, and mild obstructive sleep apnea. Since she expressed a strong will to stop eating at night, we proposed that she discontinue brotizolam, which she had been taking for the past 5 years. After fully explaining the risk of withdrawal symptoms, including rebound insomnia and other physical symptoms, we discontinued brotizolam and started her on suvorexant 20 mg. On the day she switched her medication, she experienced frequent nighttime awakenings and nightmares. Moreover, strong malaise and fatigue appeared the next day. Four days later, the primary psychiatrist recommended reducing the suvorexant dose. Therefore, she took 10 mg of suvorexant thereafter. After switching from brotizolam to suvorexant, abnormal nocturnal behavior completely disappeared.

**Figure 1:**
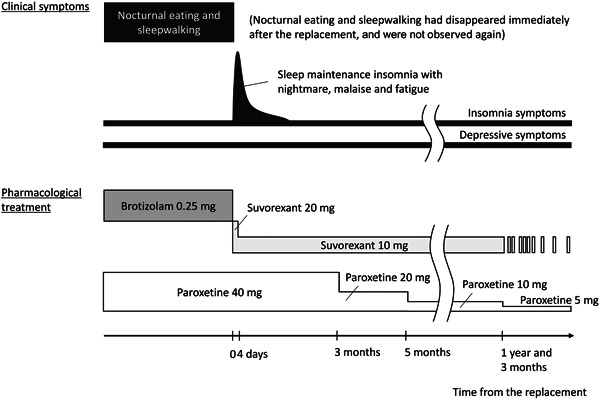
Clinical course of the present case. The patient's nocturnal eating and sleepwalking disappeared after switching from brotizolam to suvorexant and did not recur for at least 2 years. Subsequently, she experienced sleep maintenance problems and severe fatigue suspected to result from benzodiazepine withdrawal. However, these symptoms were resolved within 1 month.

For 1 month after the switch, the insomnia symptoms and fatigue gradually improved. She experienced nocturnal awakenings once daily and could not reenter sleep; however, no exacerbation of depressive symptoms was observed. Paroxetine was tapered by her primary psychiatrist to 20 mg 3 months after the switch and to 10 mg 2 months later without relapse of mood symptoms. One year and 3 months after switching to suvorexant, her paroxetine dose was reduced to 5 mg. Around that time, she used suvorexant only occasionally but could sleep reasonably well at night (Figure 1). She has not shown any abnormal nighttime behavior for at least 2 years and lost 7 kg of weight compared to her initial visit to our clinic.

## DISCUSSION

To the best of our knowledge, this is the first case report demonstrating the efficacy of orexin receptor antagonists for SRED. In the present case, nocturnal eating and sleepwalking disappeared completely after the patient switched from brotizolam to suvorexant, independent of changes in mood or lifestyle. The improvement in SRED symptoms in this case may not have resulted from the effectiveness of the orexin receptor antagonist itself but rather from discontinuation of the benzodiazepine brotizolam. The possible benefit of benzodiazepine discontinuation on reduced nocturnal eating is consistent with our previous report.^10^ Although the reason why suvorexant does not worsen nocturnal eating behavior or sleepwalking is unclear, it may be because suvorexant does not cause memory impairment,^13^ unlike GABA_A_ receptor agonists.^7^ Additionally, suvorexant does not affect the arousal threshold,^14^ thus being less likely to cause sleep inertia after nocturnal awakening. The present patient's sleep‐disordered breathing, although mild, could also have induced SRED/sleepwalking through sleep fragmentation along with brotizolam use. Benzodiazepines can cause withdrawal symptoms, including insomnia and physical symptoms, when discontinued abruptly.^15^, ^16^ In the present case, the rapid switch to suvorexant was followed by rebound insomnia and fatigue, which were considered to be withdrawal symptoms. Nightmares are a common side‐effect of suvorexant,^17^ but considering that they resolved despite continued suvorexant use, they could be a withdrawal symptom.^15^ Despite the varied withdrawal symptoms, suvorexant helped alleviate insomnia symptoms^11^ allowing the patient to continue treatment without restarting brotizolam. Providing adequate information about withdrawal symptoms^16^ would have been helpful. The immediate disappearance of SRED and sleepwalking may also have motivated the patient.

Selective serotonin reuptake inhibitors are effective for SRED.^18^, ^19^, ^20^ Therefore, we cannot rule out the possibility that paroxetine may have been protective against the symptoms of SRED as well as the recurrence of depression.^21^ Furthermore, the role of suvorexant is limited to its adjunctive effect on insomnia symptoms, and whether or not suvorexant will be effective in patients with SRED not taking benzodiazepines is unknown. More studies with a large sample size are required to clarify the effect of orexin receptor antagonists on idiopathic SRED.

## CONCLUSION

Nocturnal eating and sleepwalking in patients with SRED have been successfully treated through benzodiazepine discontinuation and suvorexant use. The present case suggests that suvorexant may be useful for SRED, at least as a sleep aid that does not stimulate nocturnal eating behavior. However, further studies are required to confirm this preliminary finding.

## AUTHOR CONTRIBUTIONS

Kentaro Matsui led the study, conducted research, and wrote the original draft. Ayano Kimura provided resources and refined the manuscript. Kentaro Nagao and Takuya Yoshiike contributed to research design, with Takuya also editing the manuscript. Kenichi Kuriyama helped shape the study and managed the project.

## CONFLICT OF INTEREST STATEMENT

The authors declare no conflicts of interest.

## ETHICS APPROVAL STATEMENT

This study was conducted according to the principles of the Declaration of Helsinki.

## PATIENT CONSENT STATEMENT

Written informed consent for presentation of her clinical course was given by the patient.

## CLINICAL TRIAL REGISTRATION

N/A.

## Data Availability

The data that support the findings of this study are available from the corresponding author upon reasonable request, subject to the necessary de‐identification processes and obtaining the appropriate patient consent.
